# β-CD-Induced Precipitation of Eriochrome Black T Recovered via CTAB-Assisted Foam Fractionation for Adsorption of Trace Cu(II)

**DOI:** 10.3390/molecules28124619

**Published:** 2023-06-07

**Authors:** Yunkang Chang, Chengsong Cao, Yuhuan Li, Yitong Yin, Yangjing Liu, Rui Li, Yimin Zhu

**Affiliations:** 1 Institute of Environmental Remediation, Dalian Maritime University, Dalian 116026, China; cyk@dlmu.edu.cn; 2 School of Biological Science, Jining Medical University, Rizhao 276826, China

**Keywords:** Eriochrome black T, separation, β-CD-CTAB-EBT particles, reuse, Cu(II) ions

## Abstract

In order to remove and reuse the ecotoxic dye Eriochrome black T (EBT) from dyeing wastewater, we used a process called cetyltrimethylammonium bromide (CTAB)-assisted foam fractionation. By optimizing this process with response surface methodology, we achieved an enrichment ratio of 110.3 ± 3.8 and a recovery rate of 99.1 ± 0.3%. Next, we prepared composite particles by adding β-cyclodextrin (β-CD) to the foamate obtained through foam fractionation. These particles had an average diameter of 80.9 μm, an irregular shape, and a specific surface area of 0.15 m^2^/g. Using these β-CD-CTAB-EBT particles, we were able to effectively remove trace amounts of Cu^2+^ ions (4 mg/L) from the wastewater. The adsorption of these ions followed pseudo-second-order kinetics and Langmuir isotherm models, and the maximal adsorption capacities at different temperatures were 141.4 mg/g at 298.15 K, 143.1 mg/g at 308.15 K, and 144.5 mg/g at 318.15 K. Thermodynamic analysis showed that the mechanism of Cu^2+^ removal via β-CD-CTAB-EBT was spontaneous and endothermic physisorption. Under the optimized conditions, we achieved a removal ratio of 95.3 ± 3.0% for Cu^2+^ ions, and the adsorption capacity remained at 78.3% after four reuse cycles. Overall, these results demonstrate the potential of β-CD-CTAB-EBT particles for the recovery and reuse of EBT in dyeing wastewater.

## 1. Introduction

As society has developed rapidly, the consumption of dyes in various industries such as textiles, printing, dyeing, and medicine has increased [1]. However, many dyes have low fixation ability, meaning that they have to be discharged with a large volume of wastewater at the end of the production process [2,3]. This dye wastewater can cause serious environmental pollution, making its treatment a significant issue in the field of environmental protection [4]. One commonly found dye in wastewater is Eriochrome black T (EBT), an acidic and reactive dye with a conjugated azo (-N=N-) group as a chromophore [5,6]. EBT can harm the environment and aquatic organisms, as well as pose a risk to human health [7,8]. It is also resistant to biological treatment [9,10], making it important to find effective methods for removing EBT from water bodies.

Currently, there are two main strategies for removing EBT from dye wastewater: degradation and separation [3]. Fenton oxidation and photocatalytic oxidation are commonly used for degrading EBT [11,12,13], with the latter being preferred due to its minimal environmental impact [14]. However, the low light transmittance of dyeing wastewater can reduce the efficiency of photocatalytic degradation [15], and recovering nano-photocatalysts from wastewater can also be challenging [16]. As an alternative, separating EBT from dye wastewater for reuse may be a better option. Adsorption separation has been widely applied to remove EBT from simulated wastewater in the laboratory [17,18,19], but real wastewater often contains surfactants [20] that can interact with dyes, especially those with opposite charges, and decrease their efficiency in adsorption separation [21]. Foam fractionation may be a cost-effective separation method for removing EBT in the presence of surfactants, as these agents help to adsorb dye molecules at the gas–liquid interface [22]. However, there have been few reports on the further treatment of recovered surfactant–dye complexes, possibly because they have more complex structures than dyes alone [23,24]. This lack of research means that the application of foam fractionation for removing EBT from dye wastewater containing surfactants is still largely limited.

In a previous study, we discovered that cetyltrimethylammonium bromide (CTAB)-EBT complexes can be precipitated via β-cyclodextrin (β-CD) to form insoluble particles [25]. Additionally, EBT and β-CD have been shown to interact with Cu(II) ions [26,27]. Based on this information, we aimed to use CTAB-assisted foam fractionation to separate EBT from dye wastewater and then use β-CD to precipitate the recovered CTAB-EBT complexes and finally utilize the insoluble particles to remove Cu(II) ions from wastewater through adsorption. In this way, we hoped to overcome the limitations of using foam fractionation to treat EBT-containing wastewater. More importantly, the costs of the wastewater treatment will be reduced effectively by such a cost-effective separation method and the reuse of EBT. This study is divided into three main sections: (1) optimizing the foam fractionation of EBT in the presence of CTAB using response surface methodology (RSM) with a Box–Behnken design (BBD) approach, a statistical experimental design technique used in the field of optimization and process improvement [28]; (2) preparing and characterizing β-CD-CTAB-EBT particles; and (3) using β-CD-CTAB-EBT particles to remove Cu(II) ions through adsorption.

## 2. Results and Discussion

### 2.1. Optimization of CTAB-Assisted Foam Fractionation of EBT Using RSM

To optimize the CTAB-assisted foam fractionation (FF) of EBT for effective recovery from simulated wastewater, we used a Box–Behnken design for the RSM with four independent variables: volumetric air flow rate (*A*), liquid loading volume (*B*), CTAB-EBT molar ratio (*C*), and pH (*D*). The response items were the enrichment ratio (*E_f_*, *Y*_1_) and recovery percentage (*R_f_*, *Y*_2_) of EBT. Each variable had three levels, resulting in a total of 27 experimental runs, the results of which are shown in Table 1.

The data in Table 1 were used to fit the dependency of both response items on the four independent variables into a quadratic regression model. The resulting polynomial equations, in terms of the coded factors shown in Table 2, are given in Equations (1) and (2).

*Y*_1_ = 75.43 − 24.58*A* + 30.43*C* − 45.85*C*^2^ (1)

*Y*_2_ = 96.63 + 49.34*C* − 1.13*AB* + 2.1*AD* − 1.3*BD* − 46.78*C*^2^ (2)

Table 3 presents a summary of the ANOVA for the two regression models. The *F* values for both models (15.06 and 4148.97) are significant at *p* < 0.0001, indicating their high statistical significance. For *Y*_1_, variables *A*, *C*, and *C*^2^ are significant model terms (*p* < 0.0001). For *Y*_2_, variables *C*, *AB*, *AD*, *BD*, and *C*^2^ are significant (*p* < 0.05). The *p* values for “lack of fit” are 0.1401 and 0.7613, which are higher than 0.05, indicating that they are non-significant relative to pure error and thus confirm the goodness of fit [29]. The determination coefficients (R2) for *Y*_1_ and *Y*_2_ (0.9461 and 0.9998, respectively) indicate that there is only a 5.49% and 0.02% chance that the models with four factors in the selected ranges cannot explain the variations in *Y*_1_ and *Y*_2_, respectively [30]. The adjusted R2 values for *Y*_1_ and *Y*_2_ (0.8833 and 0.9996, respec-tively) are close to the corresponding R2 values, suggesting that both regression models can effectively explain the variations in the response items. The model for *Y*_2_ has a par-ticularly good fit. The statistical validity of the developed models is confirmed by cal-culating the coefficients of variation (CV), with lower values indicating more signifi-cant models [31]. As shown in Table 3, the CV for both models is low (22.75% and 1.15%, respectively), indicating the accuracy of the experimental results. In addition, the precision of both models is higher than 4 (12.368 and 154.634, respectively), indi-cating an appropriate signal-to-noise ratio [32]. Based on these results, we conclude that both models can be used to design experiments.

**Figure 1:**
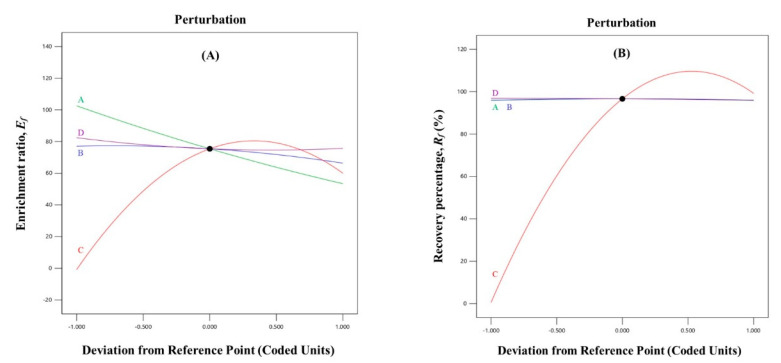
Perturbation plots that show the trends in *E_f_* (**A**) and *R_f_* (**B**) of EBT on volumetric air flow rate—*A*, liquid loading volume—*B*, CTAB-EBT molar ratio—*C*, and pH—*D* obtained at volumetric air flow rate of 250 mL/min, liquid loading volume of 400 mL, CTAB-EBT molar ratio of 1:1, and pH of 7.0.

The perturbation plots in Figure 1 demonstrate the influence of the independent variables on the *E_f_* and *R_f_* of EBT at a specific point in the design space.

The sensitivity of the response variables to a particular factor is typically proportional to the slope (curvature) of the plot [33]. In general, the CTAB-EBT molar ratio had the greatest impact on both response variables. As the CTAB-EBT molar ratio increased, the enrichment ratio and recovery of EBT also increased, reached a maximum, and then decreased. These results suggest that high values of both response variables could be achieved simultaneously at the same CTAB-EBT molar ratio. In addition, the volumetric air flow rate (*A*) and liquid loading volume (*B*) slowly decreased the *E_f_* and had a negligible effect on the *R_f_*. Furthermore, the pH of the solution (*D*) did not significantly affect either of the response variables. Using the enrichment ratio, it was possible to determine optimal values for the volumetric air flow rate, liquid loading volume, and pH.

To optimize the experimental conditions for CTAB-assisted foam fractionation, we considered both the *E_f_* and *R_f_* of EBT using the Design Expert software. The best performance was predicted using a volumetric air flow rate of 101 mL/min, a liquid loading volume of 447 mL, a CTAB-EBT molar ratio of 1.1:1, and a pH of 5.0, resulting in an enrichment ratio of 108.5 and an EBT recovery of 106.3%. To confirm these predictions, we conducted experiments under the proposed conditions in triplicate and found that the enrichment ratio was 110.3 ± 3.8 and the EBT recovery was 99.1 ± 0.3%, which are close to the predicted values.

### 2.2. Characterization of β-CD-CTAB-EBT Particles

#### 2.2.1. Morphology of β-CD-CTAB-EBT Particles

**Figure 2:**
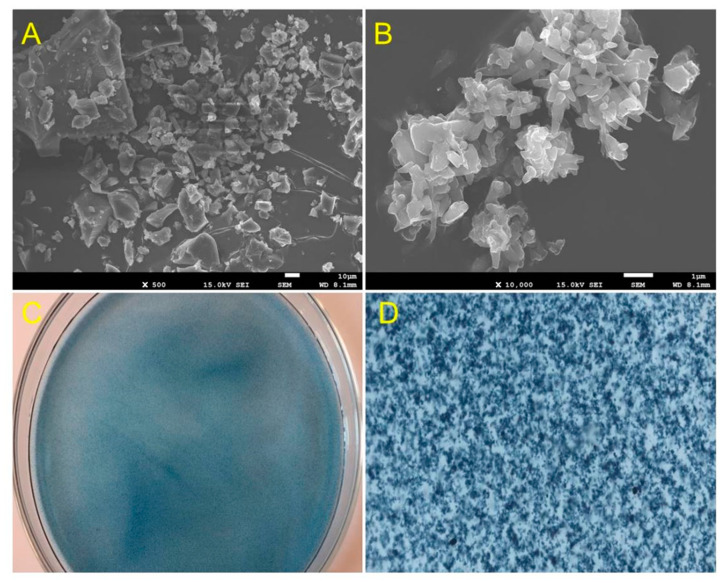
SEM images of β-CD-CTAB-EBT particles ((**A**) amplification × 500 and (**B**) amplification × 10,000) and optical images of β-CD-CTAB-EBT particles dispersed in water ((**C**) without amplification and (**D**) amplification × 40).

The CTAB-EBT mixture was obtained via foam fractionation using the experimental conditions described in Section 2.1. β-CD was then combined with this foamate to prepare β-CD-CTAB-EBT particles using the procedure described in Section 3.4. SEM images in Figure 2A,B show that the β-CD-CTAB-EBT particles had an irregular shape due to the accumulation of irregular cones. Additionally, when dispersed in water, the particles appeared flocculent (see Figure 2C,D).

#### 2.2.2. Size and Zeta Potential of β-CD-CTAB-EBT Particles

**Figure 3:**
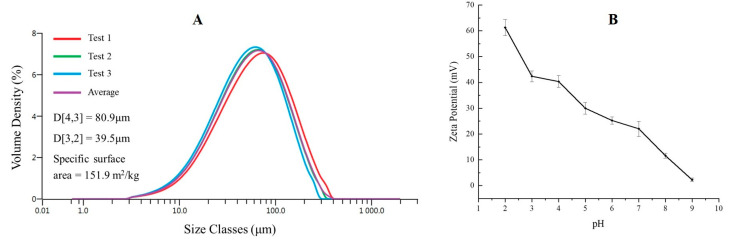
The results of three consecutive measurements of β-CD-CTAB-EBT particles size distribution with calculated particle size parameter (**A**), and variation in the zeta potential of particles with pH (**B**).

To gain further insights into the properties of the novel β-CD-CTAB-EBT particles, the size distribution and zeta potential in water were analyzed. As shown in Figure 3A, the particle size ranged from 2.59 μm to 336 μm with a volume density between 0.01 and 7.45%. The most abundant particles had a diameter of 66.4 μm and a volume density of 7.45%. Using these data, the mean volume-related equivalent diameter (D[4,3]) and the mean surface-area-related equivalent diameter (D[3,2]) were calculated via Mastersizer 3000 and found to be 80.9 μm and 39.5 μm, respectively [33]. The mean specific surface area of the particles was also determined to be 0.15 m^2^/g. The large differences in particle size and the wide particle size distribution are consistent with their irregular shape [34].

The charge of an adsorbent plays a crucial role in the adsorption of metal cations from a solution [35]. Therefore, we measured the zeta potential of the β-CD-CTAB-EBT particles in a pH range from 2.0 to 9.0. The results in Figure 3B show that the zeta potential decreases from 61.3 ± 3.1 mV to 2.2 ± 0.6 mV as the pH value increases. The positive zeta potential at all pH values suggests that the β-CD-CTAB-EBT particles are positively charged and may have electrostatic repulsion with Cu(II), which could potentially limit their adsorption efficiency.

#### 2.2.3. SEM-EDS Analysis of Cu(Ⅱ) Ions@β-CD-CTAB-EBT Particles

**Figure 4:**
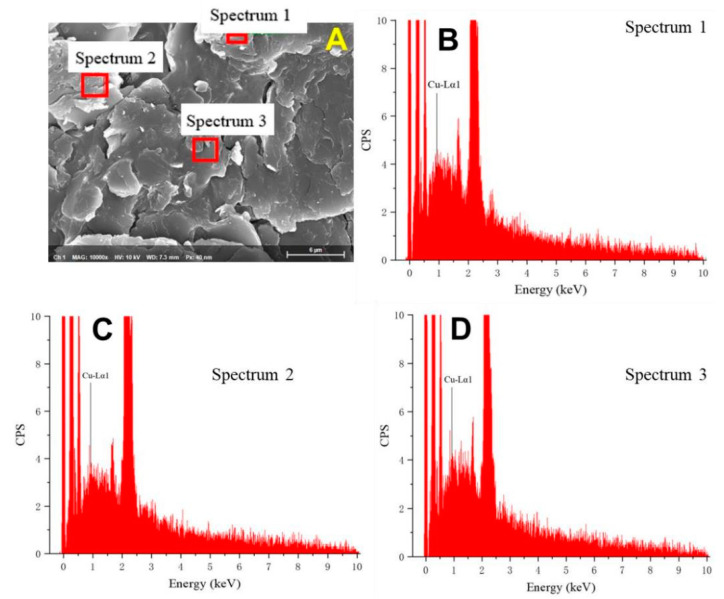
SEM image of scanning sites for EDS (**A**) and corresponding spectra of Cu(Ⅱ) ions@β-CD-CTAB-EBT particles (**B**–**D**).

We performed SEM-EDS analyses of three random sites on the Cu@β-CD-CTAB-EBT surface to confirm the adsorption of Cu^2+^ ions. The results, shown in Figure 4, indicate the presence of copper at all examined sites. This suggests that β-CD-CTAB-EBT can be used for the adsorption of Cu^2+^, even though it has a positive zeta potential at the pH of the experiment (pH = 4.0).

### 2.3. Adsorption of Cu(Ⅱ) Ions Using β-CD-CTAB-EBT Particles

#### 2.3.1. Adsorption Kinetics

**Figure 5:**
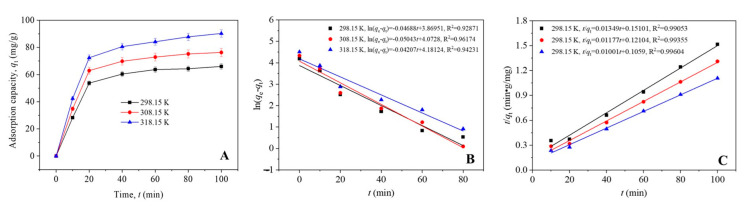
The changes in the adsorption capacity of β-CD-CTAB-EBT particles for Cu(II) ions over time at 298.15, 308.15, and 318.15 K (**A**). The data are also plotted with linear fits according to the PFO kinetics model (**B**) and the PSO kinetics model (**C**).

To study the kinetics of Cu^2+^ adsorption at the surface of β-CD-CTAB-EBT particles, we monitored the time-dependent changes in the adsorption capacity at three temperatures (298.15, 308.15, and 318.15 K). As shown in Figure 5A, the adsorption capacity increased quickly from 0 to 20 min, and then slowly approached equilibrium from 20 to 100 min.

Additionally, higher temperatures increased the adsorption of Cu^2+^ ions. We then fitted these data to PFO and PSO kinetics models to determine which model better describes the adsorption kinetics. The results in Figure 5B,C show that both models are statistically significant (*p* < 0.05), but the correlation coefficients were higher for the PSO model. Furthermore, the ANOVA results of linear fitting showed that the *F* values for the PSO model fitting (523.8 at 298.15 K, 771.2 at 308.15 K, and 1257.8 at 318.15 K) were much higher than those for the PFO model fitting (66.1 at 298.15 K, 126.7 at 308.15 K and 82.7 at 318.15 K). It is indicated that *t*/*q_t_* vs. *t* was more likely to be linear than ln(*q_e_* − *q_t_*) vs. *t*. Therefore, the PSO model better described the variation in the adsorption capacity of Cu(II) at β-CD-CTAB-EBT particles with time. According to this model, the equilibrium capacities of β-CD-CTAB-EBT particles in a 4 mg/L Cu(II) solution are 74.1 mg/g at 298.15 K, 85.0 mg/g at 308.15 K, and 99.9 mg/g at 318.15 K.

#### 2.3.2. Adsorption Isotherms

**Figure 6:**
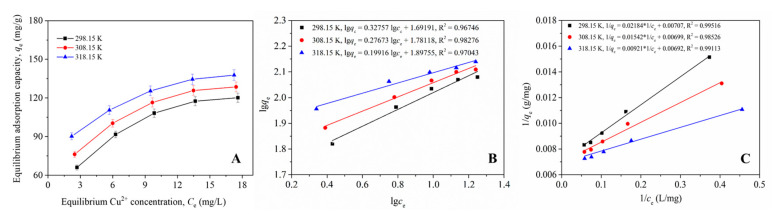
The dependence of the equilibrium adsorption capacity of β-CD-CTAB-EBT on the equilibrium concentration of Cu^2+^ in aqueous solution is shown at three temperatures: 298.15, 308.15, and 318.15 K (**A**). The data are also plotted with fits to the Freundlich adsorption isotherm (**B**) and the Langmuir adsorption isotherm (**C**).

We examined the effect of temperature and starting concentration of Cu^2+^ ions on the equilibrium adsorption capacity (*q_e_*) of β-CD-CTAB-EBT. As shown in Figure 6A, the *q_e_* increased with the concentration of Cu^2+^ ions in the solution, and this increase was more pronounced at lower concentrations. In addition, temperature increased *q_e_* at each equilibrium Cu(II) concentration. To gain a better understanding of the adsorption process, the data from Figure 6A were fitted to Langmuir and Freundlich models.

The results in Figure 6B,C show that both models are statistically significant (*p* < 0.05). Thus, both Langmuir and Freundlich models could be used to explain the results in Figure 6A. The ANOVA results of linear fitting presented that the *F* values for the Langmuir model fitting were 823.1, 268.4, and 448.0 at 298.15, 308.15, and 318.15 K, higher than those for the Freundlich model fitting (119.9, 229.4, and 132.3, respectively). Thus, 1/*q_e_* vs. *C_e_* had higher probabilities in linear relationships than lg*q_e_* vs. lg*C_e_*. Furthermore, the Langmuir model had a higher correlation coefficient at each temperature. This suggests that Cu(II) likely adsorbs at the surface of β-CD-CTAB-EBT particles by forming a single layer. Based on the Freundlich model, the maximal adsorption capacities at 298.15, 308.15, and 318.15 K were calculated as 141.4, 143.1, and 144.5 mg/g, respectively.

#### 2.3.3. Adsorption Thermodynamics

The thermodynamic parameters of the adsorption of Cu^2+^ ions onto β-CD-CTAB-EBT particles were calculated based on the temperature changes in the *q_e_* vs. *C_e_* profiles (Figure 6A).

As shown in Table 4, Δ*G* had negative values at all temperatures, indicating that the process is spontaneous [36]. Additionally, the positive values of Δ*H* indicate that the adsorption is endothermic [37]. The positive Δ*S* values of 0.37 kJ/(mol·K) suggest that the randomness of the system slightly increases upon the adsorption of Cu(II) ions [36]. These observations suggest that the interactions between Cu^2+^ and the β-CD-CTAB-EBT surface obey the laws of physisorption [38] and β-CD and EBT were responsible for the adsorption of Cu^2+^. Their interactions with Cu^2+^ may be driven by Van der Waals forces, covalent binding, or surface precipitation because of unfavorable electrostatic interactions between cations and the positively charged adsorbent surface [26,27,39].

#### 2.3.4. Effect of pH on the Removal Ratio of Cu^2+^ Ions and Recyclability of β-CD-CTAB-EBT Particles

**Figure 7:**
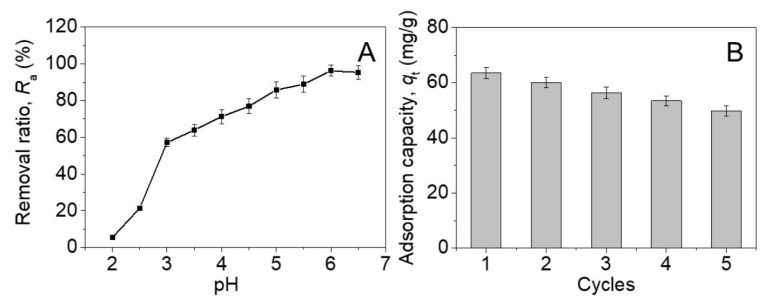
The effect of pH on removal ratio of Cu^2+^ (**A**) and recyclability of β-CD-CTAB-EBT particles (**B**).

The positive charge of β-CD-CTAB-EBT particles at low pH (Figure 3A) allows for the easy desorption of physically adsorbed copper cations from the surface, enabling the reuse of the adsorbent. The effect of pH on the adsorption efficiency of Cu(II) ions was investigated by changing the pH value from 2.0 to 6.5 while keeping the other parameters constant. As shown in Figure 7A, the Cu^2+^ removal ratio increased from 5.4 ± 0.5% at pH 2.0 to 95.3 ± 3.0% at pH 6.0, with little additional change at pH 6.5. This trend may be attributed to the decreased amount of positive charge on the surface of β-CD-CTAB-EBT particles at higher pH values (Figure 3B), which allows for stronger interactions with copper ions and therefore a higher removal efficiency.

To recycle the β-CD-CTAB-EBT adsorbent, we used a 10 mM solution of sulfuric acid to desorb Cu(II) ions. The initial adsorption capacity (*q_e_*) of β-CD-CTAB-EBT for Cu^2+^ ions was 63.5 ± 2.0 mg/g (Figure 7B). After five consecutive cycles of reuse, the *q_e_* decreased by 21.7% to 49.7 ± 1.9 mg/g. Despite this decrease, the β-CD-CTAB-EBT particles demonstrated a relatively high and reversible adsorption capacity, making them a promising candidate for the removal of Cu^2+^ from wastewater. In a word, EBT was effectively separated and reused by the CTAB-assisted foam fractionation and subsequent β-CD-induced precipitation. However, there was still a common drawback for this technology that the treatment of by-products after the adsorption of Cu^2+^ ions would need lots of effort.

## 3. Materials and Methods

### 3.1. Chemicals

The *analytical-grade* EBT, CTAB, and β-CD were ordered from Sinopharm (Beijing, China). EBT was dissolved in a 10 mM Na_2_HPO_4_-H_3_PO_4_ solution at pH 7.0 to a concentration of 0.22 mM. A 6 M H_3_PO_4_ or NaOH solution was used to adjust the pH of each solution, which was measured using a PHS-3C pH meter (Shanghai Yifen Scientific Instrument Co., Ltd., Shanghai, China). Ultrapure water (18.2 MΩ) was used in all experiments.

### 3.2. Equipment for EBT Batch Foam Fractionation

**Figure 8:**
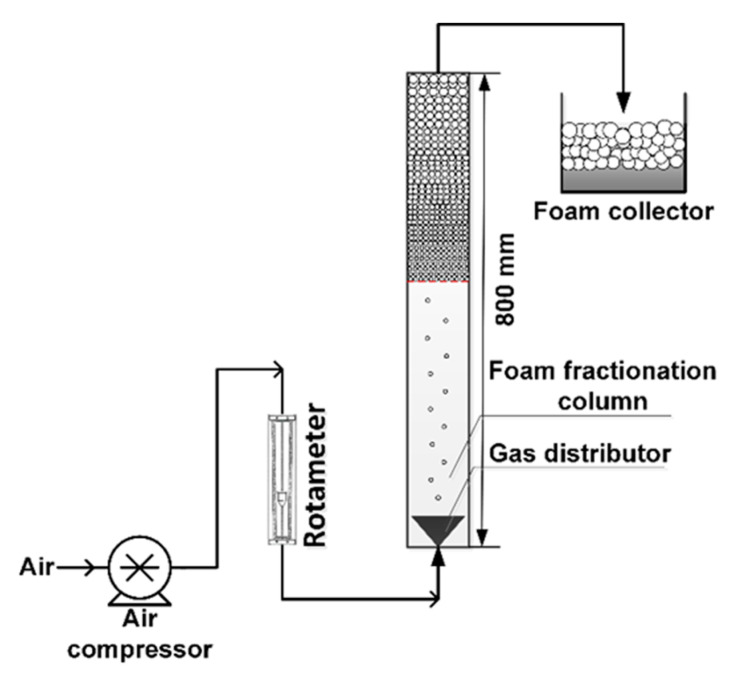
Diagram that illustrates batch foam fractionation of EBT.

A simulated dye wastewater containing surfactant was prepared by mixing the EBT solution with a certain amount of CTAB. The CTAB-EBT mixture was then separated from this wastewater using batch foam fractionation (FF) equipment, as shown in Figure 8.

The FF column was made of transparent plexiglass and had dimensions of 800 mm × 50 mm (height × inner diameter). A gas distributor with a mean pore diameter of 180 μm was placed at the exit of the column. An air compressor (ACO-018A from Guangdong Haili Group Co., Ltd., in Rudong, China) was used to pump air through the column at a flow rate between 60 and 600 mL/min that was controlled using an air rotameter (LZB-W from Shanghai Automation Instrument Co., Ltd., in Shanghai, China). The resulting foam was stored for further use. The performance of the FF process was studied by calculating the enrichment ratio (*E_f_*) and recovery percentage (*R_f_*) using Equations (3) and (4), respectively.

(3) $$E_{f}=\frac{A_{f}}{Ao}$$

(4) $$R_{f}=\frac{V_{f}A_{f}}{V_{o}Ao}\times 100\%$$

where *A_f_* and *A_o_* are absorbances of the foamate and the feed solution, respectively, measured using a colorimeter (CN60M/SD9011, Westmid, Birmingham, UK); *V_f_* and *V_o_* are the volumes of the foamate and the feed solution (in mL).

### 3.3. Optimization of EBT Batch Foam Fractionation via RSM

Volumetric air flow rate, liquid loading volume, surfactant–dye molar ratio, and pH have been shown to significantly affect the surfactant-assisted foam fractionation of dyes [40,41]. Therefore, we used a Box–Behnken design of the response surface methodology (BBD-RSM) to optimize the CTAB-assisted foam fractionation of the EBT mixture, with these four factors as independent variables at three levels, and the *E_f_* and *R_f_* as response items. The starting points for the optimization were the results of single-factor experiments (not presented here). The specific levels of the four factors used in the optimization are listed in Table 1, and Table 2 shows the base run of 27 experiments designed using the software Design Expert (Version 13.0.1.0. Stat-Ease Inc., Minneapolis, MN, USA) and the corresponding results.

### 3.4. Preparation of β-CD-CTAB-EBT Particles

**Figure 9:**
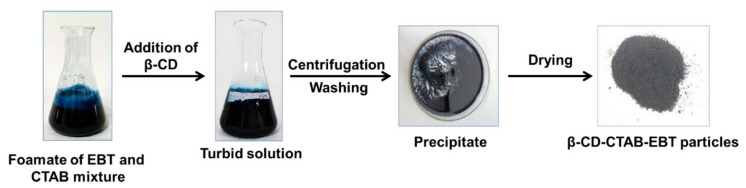
Process flow diagram for the preparation of β-CD-CTAB-EBT particles from the foamate of EBT and CTAB mixture.

The preparation of β-CD-CTAB-EBT particles is depicted in Figure 9. The process begins by adding β-CD to the foamate in a molar ratio of EBT to β-CD of 1:1.2, resulting in the formation of β-CD-CTAB-EBT particles. These particles are then separated from the turbid solution through centrifugation at 5000 rpm for 10 min. The particles are subsequently washed 3× with ultrapure water and dried at 60 degrees Celsius to complete the process.

### 3.5. Adsorption Removal of Cu(Ⅱ) Ions Using β-CD-CTAB-EBT Particles

#### 3.5.1. Evaluation of Adsorption Performance

In the adsorption experiments of Cu^2+^ ions using β-CD-CTAB-EBT particles, a batch mode was used with a water area oscillator (SHA-B, Shanghai Enyi Medical Technology Development Co., Ltd., Shanghai, China). The effectiveness of the adsorption process was assessed through the calculation of the adsorption capacity (*q_t_*, mg/g) and removal ratio (*R_a_*) using Equations (5) and (6), respectively.

(5) $$q_{t}=\frac{(c_{0}-c_{t})\times V}{m}$$

(6) $$R_{a}=\frac{c_{0}-c_{t}}{c_{0}}\times 100\%$$

The Cu^2+^ ion concentration in the feed and residual solution after adsorption (in mg/L) is represented by *c*_0_ and *c_t_*, respectively. The volume of the feed solution (in L) is represented by *V*, and the mass of the added β-CD-CTAB-EBT particles (in g) is represented by *m*.

#### 3.5.2. Adsorption Kinetics

In the experimental setup, 20 mg of β-CD-CTAB-EBT particles was added to a 1000 mL copper sulfate solution with a Cu^2+^ ion concentration of 4 mg/L and a pH of 6.0. This mixture was placed in a 1000 mL conical flask and placed in a water area oscillator, which was set to shake at 200 rpm at three temperatures (298.15, 308.15, and 318.15 K). Aliquots of the mixture (2 mL) were taken at intervals of 10 min, and the β-CD-CTAB-EBT particles were removed by filtering through a 0.22 μm cellulose acetate filter mounted on a glass syringe. The concentration of Cu^2+^ ions in the filtrate was measured, and the corresponding adsorption capacity (*q_t_*) was calculated. The kinetics of the adsorption of Cu^2+^ ions at the β-CD-CTAB-EBT particles were evaluated based on the variation in *q_t_* over time using pseudo-first-order (PFO) and pseudo-second-order (PSO) models, as defined in Equations (7) and (8), respectively [42].

(7) $$\ln(q_{e}-q_{t})=\ln q_{e}-k_{1}t$$

(8) $$\frac{t}{q_{t}}=\frac{1}{k_{2}q_{e}^{2}}+\frac{t}{q_{e}}$$

In these equations, *q_e_* (mg/g) represents the equilibrium absorption capacity of Cu(II) ions, *t* (min) represents time, and *k*_1_ (1/min) and *k*_2_ (L/(mol × min)) represent the PFO and PSO rate constants, respectively.

#### 3.5.3. Adsorption Isotherms

The experimental procedures in this section were similar to those described in Section 3.5.2, with the exception that the starting concentrations of Cu^2+^ ions were set at 4, 8, 12, 16, and 20 mg/L. In each experiment, *q_e_* and the equilibrium concentration of Cu^2+^ ions (*C_e_*, mg/L) in the residual solution were calculated by fitting the data to the PSO model. The Langmuir and Freundlich isotherm adsorption models were then used to evaluate the adsorption of Cu(II) ions in the β-CD-CTAB-EBT particles using the data of *q_e_* vs. *C_e_*. These models are represented by Equations (9) and (10), respectively [43].

(9) $$q_{e}=\frac{q_{\max}k_{L}C_{e}}{1+k_{L}C_{e}}\Rightarrow \frac{1}{q_{e}}=\frac{1}{q_{\max}k_{L}}*\frac{1}{c_{e}}+\frac{1}{q_{\max}}$$

(10) $$q_{e}=k_{F}C_{e}^{1/n}\Rightarrow \mathrm{lg}q_{e}=\mathrm{lg}k_{F}+\frac{1}{n}\mathrm{lg}c_{e}$$

In these equations, *q*_max_ (mg/g) denotes the theoretical maximal absorption capacity of Cu(II) ions, *k_L_* (L/mg) represents the Langmuir adsorption constant, *k_F_* represents the Freundlich adsorption constant, and *n* is a dimensionless coefficient.

#### 3.5.4. Adsorption Thermodynamics

The thermodynamics of the adsorption of Cu^2+^ ions at the β-CD-CTAB-EBT particles was analyzed by calculating the Δ*G*, Δ*H*, and Δ*S* values. The data obtained in Section 3.5.3 were fitted into the Van’t Hoff and the Gibbs free energy equation, as defined in Equations (11) and (12), respectively [44].

(11) $$\ln k_{d}=\frac{-\Delta H}{RT}+\frac{\Delta S}{R}$$

(12) ΔG=ΔH−TΔS

In these equations, *k_d_* represents the distribution coefficient for the Cu(II) ions adsorption, as defined in Equation (13) [45], *T* (K) is temperature, and *R* (8.314 J/(mol·K)) represents the molar gas constant.

(13) $$k_{d}=\frac{q_{e}}{C_{e}}\times 1000$$

### 3.6. Characterization of β-CD-CTAB-EBT Particles

The size distribution and zeta potential of β-CD-CTAB-EBT particles were determined using the Mastersizer 3000 and Zetasizer Pro instruments from Malvern Panalytical (Malvern, UK). The microstructure of the particles was examined using an SEM (JSM-7610F, JEOL, Tokyo, Japan) and a high-definition electronic microscope (BC1201E, Shenzhen Bosheng Electronic Technology, Shenzhen, China). The adsorption of Cu^2+^ ions on the surface of the β-CD-CTAB-EBT particles was analyzed using a ZEISS EVO 18 SEM (Carl Zeiss Microscopy, Aalen, Germany) equipped with EDS.

### 3.7. Measurement of Cu(II) Ion Concentration

The concentration of Cu^2+^ ions in an aqueous solution was determined using a spectrophotometric detection kit for copper provided by Hangzhou Luhang Seng Science and Technology (Hangzhou, China) and a U-3900H UV/VIS spectrophotometer from Hitachi (Tokyo, Japan). A linear relationship between the absorbance at 560 nm and the concentration of copper ions was established in the range from 0 to 5 mg/L, with the calibration curve equation *A*_560_ = 0.098 *C*_cu_ + 0.0157 (*R*^2^ = 0.98702).

### 3.8. Statistical Analysis

The experiments were carried out in triplicates, and the final result was presented as the mean ± standard deviation. An analysis of variance (ANOVA) at *p* ≤ 0.05 was carried out in Microsoft Excel Professional Plus 2013 (v. 15).

## 4. Conclusions

In this work, β-CD-CTAB-EBT particles were prepared by adding β-CD to the foamate of the EBT and CTAB mixture recovered from a process of the CTAB-assisted foam fractionation of EBT and then used for the adsorption of Cu^2+^ from wastewater. These particles had an irregular shape with a specific surface area of 0.15 m^2^/g and interacted with Cu^2+^ ions via nonionic forces. The kinetics of the adsorption process followed the PSO rate law, and the data fit well with the Langmuir isotherm, suggesting that the main interaction between the sorbent and sorbate was physisorption. The *q_e_* increased from 141.4 mg/g to 144.5 mg/g with an increase in temperature from 298.15 K to 318.15 K, and the process was spontaneous and endothermic. Under the optimized conditions, the removal ratio of copper ions reached 95.3%, and 78.3% of the initial *q_e_* was retained after four cycles of reuse. Thus, the β-CD-CTAB-EBT particles are a recyclable, ecofriendly material for the removal of Cu^2+^ ions from wastewater. Overall, the CTAB-assisted foam fractionation and subsequent β-CD-induced precipitation were effective in the separation and reuse of EBT. These results will have practical importance to the treatment of EBT-containing wastewater.

## Figures and Tables

**Table 1:** Experimental scheme obtained from Box–Behnken design and corresponding result data.

Run	Coded (Uncoded) Variable Level				*Y*_1_, Enrichment Ratio (*E_f_*)	*Y*_2_, Recovery Percentage (*R_f_*/*%*)
	*A*, Volumetric Air Flow Rate (mL/min)	*B*, Liquid Loading Volume (mL)	*C*, CTAB-EBT Molar Ratio	*D*, pH		
1	1 (400)	−1 (200)	0 (1)	0 (7)	45.7	95.8
2	−1 (100)	−1 (200)	0 (1)	0 (7)	107.5	94.5
3	0 (250)	−1 (200)	−1 (0.5)	0 (7)	0	0
4	0 (250)	1 (600)	1 (1.5)	0 (7)	53.9	99
5	1 (400)	0 (400)	1 (1.5)	0 (7)	35.2	98.7
6	1 (400)	1 (600)	0 (1)	0 (7)	42.9	93.2
7	−1 (100)	0 (400)	1 (1.5)	0 (7)	87.6	98.1
8	−1 (100)	0 (400)	0 (1)	1 (9)	129.5	93.7
9	0 (250)	1 (600)	0 (1)	1 (9)	58.6	93.5
10	0 (250)	0 (400)	1 (1.5)	−1 (5)	68.1	98.8
11	0 (250)	0 (400)	1 (1.5)	1 (9)	53.2	98.8
12	1 (400)	0 (400)	−1 (0.5)	0 (7)	0	0
13	1 (400)	0 (400)	0 (1)	−1 (5)	49.4	94.1
14	0 (250)	−1 (200)	0 (1)	−1 (5)	99.5	95.6
15	0 (250)	0 (400)	0 (1)	0 (7)	78.6	95.5
16	−1 (100)	0 (400)	−1 (0.5)	0 (7)	0	0
17	0 (250)	1 (600)	0 (1)	−1 (5)	74.1	98.7
18	0 (250)	1 (600)	−1 (0.5)	0 (7)	0	0
19	0 (250)	−1 (200)	1 (1.5)	0 (7)	67.1	98.7
20	0 (250)	0 (400)	0 (1)	0 (7)	69.2	96.8
21	0 (250)	0 (400)	−1 (0.5)	1 (9)	0	0
22	0 (250)	0 (400)	−1 (0.5)	−1 (5)	0	0
23	0 (250)	−1 (200)	0 (1)	1 (9)	63.5	95.6
24	−1 (100)	0 (400)	0 (1)	−1 (5)	109.7	98.1
25	−1 (100)	1 (600)	0 (1)	0 (7)	89.5	96.4
26	1 (400)	0 (400)	0 (1)	1 (9)	55.6	98.1
27	0 (250)	0 (400)	0 (1)	0 (7)	78.5	97.6

**Table 2:** Variables and their levels used for Box–Behnken design.

Variables	Label	Levels		
		−1	0	1
*A*	Volumetric air flow rate (mL/min)	100	250	400
*B*	Liquid loading volume (mL)	200	400	600
*C*	CTAB-EBT molar ratio	0.5	1	1.5
*D*	pH	5	7	9

**Table 3:** ANOVA for the response surface quadratic regression models.

Source	*Y*_1_			*Y*_2_		
	Mean Square	*F*-Value	*p*-Value	Mean Square	*F*-Value	*p*-Value
Model	2458.9	15.06	<0.0001	3117.78	4148.97	<0.0001
*A*	7252.08	44.41	<0.0001	0.0675	0.0898	0.7695
*B*	344.54	2.11	0.172	0.03	0.0399	0.845
*C*	11,108.17	68.03	<0.0001	29,215.2	38,878	<0.0001
*D*	136.01	0.8329	0.3794	2.61	3.48	0.0868
*AB*	57.76	0.3537	0.5631	5.06	6.74	0.0234
*AC*	686.44	4.2	0.0628	0.09	0.1198	0.7353
*AD*	46.24	0.2832	0.6043	17.64	23.47	0.0004
*BC*	43.56	0.2668	0.6149	0.0225	0.0299	0.8655
*BD*	105.06	0.6434	0.4381	6.76	9	0.0111
*CD*	55.5	0.3399	0.5707	0	0	1
*A* ^2^	35.59	0.218	0.649	2.64	3.52	0.0852
*B* ^2^	73.18	0.4481	0.5159	2.37	3.15	0.1011
*C* ^2^	11,213.89	68.67	<0.0001	11,670.88	15,530.98	<0.0001
*D* ^2^	71.38	0.4371	0.521	0.1481	0.1971	0.6649
Residual	163.29			0.7515		
Lack of Fit	190.12	6.52	0.1401	0.6771	0.6027	0.7613
Pure Error	29.14			1.12		
*R* ^2^	0.9461			0.9998		
Adj. *R*^2^	0.8833			0.9996		
Variation coefficient	22.75%			1.15%		
Adequate precision	12.368			154.634		

**Table 4:** Thermodynamic parameters for the adsorption of Cu (Ⅱ) ions at β-CD-CTAB-EBT particles.

T/K	Δ*G* (KJ/mol)	Δ*H* (KJ/mol)	Δ*S* (KJ/(mol T))
298.15	−31.84	78.48	0.37
308.15	−35.54		
318.15	−39.24		

## Data Availability

The data are contained within the article.
